# Application of Single-Cell Multi-Omics in Dissecting Cancer Cell Plasticity and Tumor Heterogeneity

**DOI:** 10.3389/fmolb.2021.757024

**Published:** 2021-10-15

**Authors:** Deshen Pan, Deshui Jia

**Affiliations:** Laboratory of Cancer Genomics and Biology, Department of Urology, and Institute of Translational Medicine, Shanghai General Hospital, Shanghai Jiao Tong University School of Medicine, Shanghai, China

**Keywords:** single-cell techniques, tumor heterogeneity, cellular plasticity, clone evolution, precision therapy

## Abstract

Tumor heterogeneity, a hallmark of cancer, impairs the efficacy of cancer therapy and drives tumor progression. Exploring inter- and intra-tumoral heterogeneity not only provides insights into tumor development and progression, but also guides the design of personalized therapies. Previously, high-throughput sequencing techniques have been used to investigate the heterogeneity of tumor ecosystems. However, they could not provide a high-resolution landscape of cellular components in tumor ecosystem. Recently, advance in single-cell technologies has provided an unprecedented resolution to uncover the intra-tumoral heterogeneity by profiling the transcriptomes, genomes, proteomes and epigenomes of the cellular components and also their spatial distribution, which greatly accelerated the process of basic and translational cancer research. Importantly, it has been demonstrated that some cancer cells are able to transit between different states in order to adapt to the changing tumor microenvironment, which led to increased cellular plasticity and tumor heterogeneity. Understanding the molecular mechanisms driving cancer cell plasticity is critical for developing precision therapies. In this review, we summarize the recent progress in dissecting the cancer cell plasticity and tumor heterogeneity by use of single-cell multi-omics techniques.

## Introduction

Tumor heterogeneity, including genetic heterogeneity, epigenetic heterogeneity, phenotypic and functional heterogeneity, plays essential roles in tumor progression, especially in promoting resistance to treatment and driving metastasis (Giraudeau et al., 2019; Guo et al., 2019; Hinohara and Polyak, 2019). Investigating the origin of tumor heterogeneity has been the focus of research. For example, theories of cancer stem cell, clonal evolution and cellular plasticity have been proposed to explain the origin of heterogeneity (Michor and Polyak, 2010). Previously, high-throughput sequencing techniques have been employed to classify molecular subtypes, monitor the treatment response, identify new therapeutic targets and explore the tumor heterogeneity in the field of cancer research (Choi et al., 2017; Zhang et al., 2018; Zhao et al., 2020; Gay et al., 2021). However, these techniques have not been ideal tools to investigate the intra-tumoral heterogeneity since they usually detected the average signals of mixed cell populations rather than signals of individual cells within a tissue. For example, the bulk RNA sequencing cannot distinguish the proportions of distinct cellular components within a tumor (Roma-Rodrigues et al., 2019). Also, it is hard for the bulk sequencing techniques to determine the mutation status or transcriptome of distinct cell subpopulation in the tumor ecosystem. In contrast, single-cell techniques have shown great advantages in dissecting the cellular compositions and also their molecular features (Qian et al., 2020; Liu J. et al., 2021; Arora and Pal, 2021). Moreover, there are multiple hybrid or intermediate states of cells in the tumor ecosystem, such as hybrid epithelial/mesenchymal cells (Kroger et al., 2019; Thong et al., 2020). It is also hard for the bulk sequencing technologies to identify these hybrid cells. However, single-cell techniques have provided an opportunity to unmask hybrid states of individual cells (Williamson et al., 2016; Wouters et al., 2020; Gay et al., 2021). For example, multiple hybrid states of cancer cells have been discovered in human cancers by single-cell RNA sequencing (scRNA-seq), such as hybrid EMT cells and cancer/immune cells (Gay et al., 2021). In addition, single-cell techniques have great power to identify rare cell populations (Goveia et al., 2020; Kieffer et al., 2020; Pombo Antunes et al., 2021). Finally, single-cell techniques can distinguish tumor cells from non-tumor cell components, and also infer the interactions within or across these cellular components. Therefore, single-cell techniques provide more refined molecular features of tumor tissues compared with the bulk sequencing techniques.

Since the first application of single-cell transcriptome sequencing in 2009, single-cell techniques have evolved greatly and contributed tremendously in various fields of research (Tang et al., 2009; Navin et al., 2011; Hou et al., 2012; Liu J. et al., 2020; Zhu et al., 2020). Currently, single-cell techniques have been widely used in cancer research and shed light on the molecular underpins of tumor initiation and progression. For example, single-cell techniques have been used to analyze tumors at the levels of DNA (Gawad et al., 2016), RNA (Gonzalez-Silva et al., 2020), proteome (Wu and Singh, 2015), and epigenome (Schwartzman and Tanay, 2015). Recently, spatial transcriptomics (ST) techniques have enabled high-throughput sequencing of cellular components while preserving their spatial information within the tissue (Crosetto et al., 2015).

**FIGURE 1:**
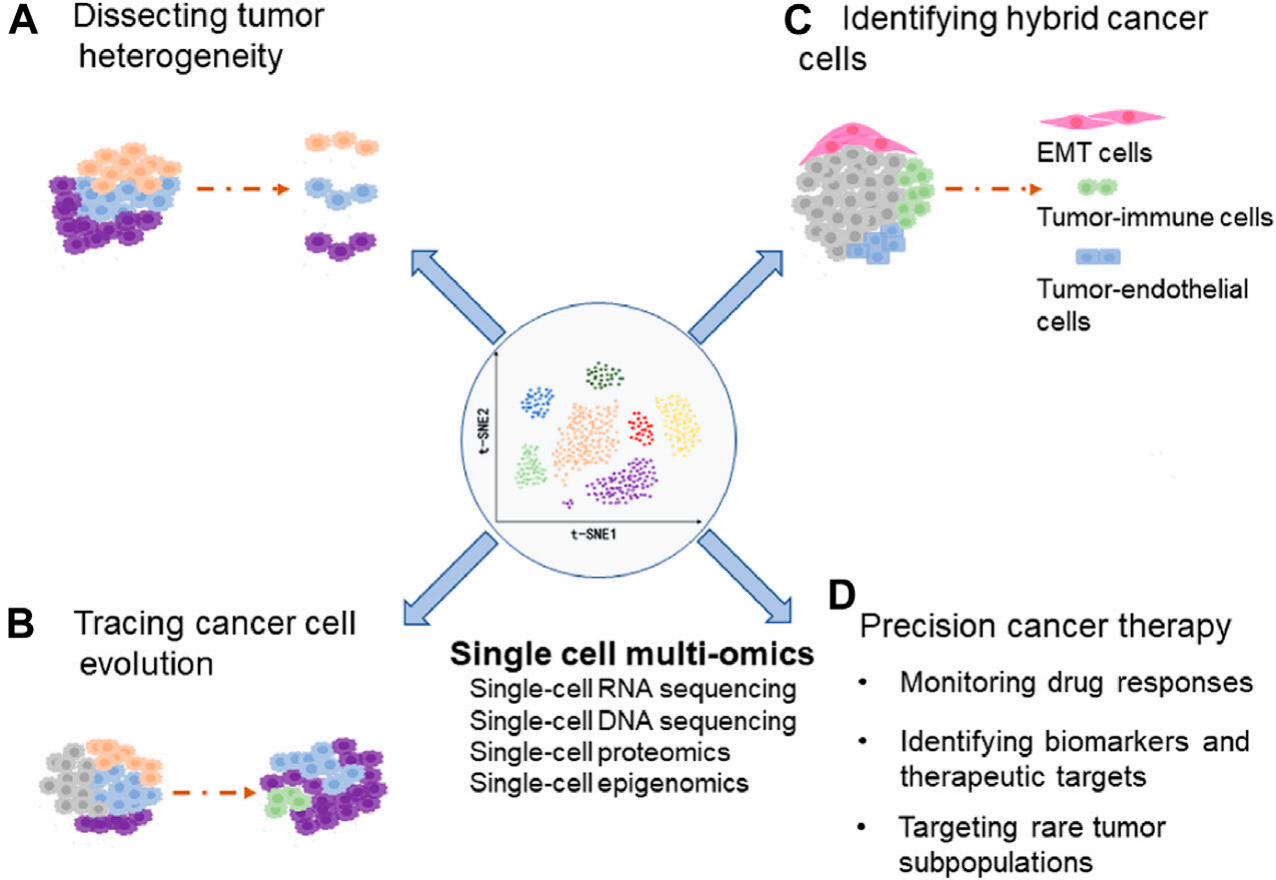
The diagram indicates applications of single-cell multi-omics in dissecting tumor heterogeneity **(A)**, tracing cancer cell evolution **(B)**, identifying hybrid cancer cells **(C)**, and precision cancer therapy **(D)**.

Cancer cell plasticity refers to some cancer cells transit dynamically between different cellular states, which results in increased tumor heterogeneity and promotes tumor progression (Gupta et al., 2011; Meacham and Morrison, 2013; Gunnarsson et al., 2020). However, molecular mechanisms that regulate cellular plasticity are still elusive. Recently, single-cell sequencing has been used to explore cancer cell plasticity (Su et al., 2017; Lourenco et al., 2020; Sacchetti et al., 2021). In this review, we summarize the recent progress in dissecting the cancer cell plasticity and tumor heterogeneity through single-cell multi-omics techniques, including the scRNA-seq, Single-Cell DNA Sequencing (scDNA-seq), single-cell proteomics and single-cell epigenomics (Figure 1).

## Dissection of Tumoral Heterogeneity

### Single-Cell RNA Sequencing Analysis of Tumor Heterogeneity

ScRNA-seq has been widely used to explore the intra-tumoral heterogeneity (Peng et al., 2019; Robertson et al., 2020; Zhang et al., 2020; Zhou et al., 2020; Wang R. et al., 2021). For example, scRNA-seq revealed seven cancer cell subpopulations in pancreatic ductal adenocarcinoma (PDAC). However, only one subpopulation was shared in most PDAC patients, whereas the other six subpopulations existed in 1–2 patients (Peng et al., 2019). Moreover, tumor cells from different PDAC patients have been found to be hardly clustered together, indicating a high inter-tumor heterogeneity of PDAC (Lin et al., 2020). Consistently, similar findings have been identified in glioblastoma (Patel et al., 2014), melanoma (Tirosh et al., 2016), and breast cancer (Karaayvaz et al., 2018). Furthermore, scRNA-seq provided a refined resolution to dissect the tumor heterogeneity (Sottoriva et al., 2013; Gay et al., 2021; Riemondy et al., 2021). ScRNA-seq has also been used to identify rare cancer cell subpopulations, which previously hardly been identified by the bulk RNA-seq. For example, five cancer cell subpopulations were identified in primary gastric adenocarcinoma by scRNA-seq, three of which corresponded to histopathological features of Lauren’s subtypes whereas the other two were recognized as new subpopulations with different molecular characteristics (Zhang M. et al., 2021). In addition, scRNA-seq explored the functional heterogeneity of distinct cancer cell subpopulations. For example, scRNA-seq identified three different transcriptional states of lung adenocarcinoma (LUAD), tS1, tS2 and tS3 (Peng et al., 2019). The transcriptional state of tS1 and tS3 were similar to that of normal lung epithelial cells, suggesting that normal lung epithelial cells may be the source of LUAD. However, the tS2 showed a completely different transcription signature, characterized by the increased expression of genes associated with advanced tumors (Tirosh et al., 2016).

### Single-Cell DNA Sequencing Analysis of Tumor Heterogeneity

ScDNA-seq has been used to identify single nucleotide variations (SNVs), copy number alterations (CNAs), and structure variants (SVs), as well as investigate the genetic heterogeneity of tumors. For instance, Duan et al. used scDNA-seq to study the CNA patterns of gastroesophageal junction cancer, and found that there are more than two subclones with different CNA patterns in both primary tumors and metastatic lymph nodes, suggesting that there is an extensive intra-tumor heterogeneity in both primary and metastatic tumors (Duan et al., 2021). Similar results have been identified in Hodgkin’s lymphoma (Mangano et al., 2019). Besides, scDNA-seq has been used to examine the heterogeneity of circulating tumor cells (CTCs) from liquid biopsies and monitor cancer genomes in a non-invasive manner. For example, scDNA-seq detected the same mutations of TP53, RB1, PIK3CA and ERBB2 genes in both biopsies and CTCs from patients with inflammatory breast cancer, suggesting that CTCs may reflect the genetic aberrations of primary tumors and act as an alternative resource of tumor heterogeneity (Bingham et al., 2017).

### Single-Cell Proteomics Analysis of Tumor Heterogeneity

Recently, single-cell proteomics techniques have also been developed to investigate tumor heterogeneity and uncover the mechanisms of tumor progression (Wagner et al., 2019; Liu L. et al., 2020; Reza et al., 2021). For example, Wagner et al. analyzed 144 human breast tumors and 50 non-tumor tissues using cytometry by time-of-flight (CyTOF). Accordingly, they classified epithelial cells into seven luminal subgroups (L1–L7) and two basal subgroups (B1 and B2). Notably, the L3 luminal subgroup was observed to express high levels of EpCAM and CD49f but low level of ERα, which are characteristics of luminal progenitor cells. In contrast, the L4 luminal subgroup was shown to express high levels of the ERα, AR, HER2, EGFR and c-MET, which are involved in tumor cell proliferation and migration. Remarkably, they found that tumors recognized as ER^+^ by Immunohistochemical staining also contain a subset of ER^−^ cell populations (Wagner et al., 2019). These findings could help to explain why nearly 30% of the ER+ breast cancers eventually develop endocrine resistance and progress to metastasis (Reinert and Barrios, 2015).

Furthermore, using RNAscope-based *in situ* hybridization protocol coupled with CyTOF, Schulz et al. have analyzed subcellular resolution mRNA and protein in breast cancer (Schulz et al., 2018). Similarly, the combination of CyTOF and immunohistochemical staining has enabled us to visualize the spatial distribution of distinct cellular compositions (Giesen et al., 2014). In addition to the CyTOF, multiple immunofluorescence imaging techniques have been used to detect multiple proteins in single cells (Lin et al., 2015; Pachynski et al., 2021). For example, cyclic immunofluorescence (CycIF) has been used to examine the formalin-fixed, paraffin-embedded (FFPE) specimens(Lin et al., 2018).

### Single-Cell Epigenomics Analysis of Tumor Heterogeneity

Single-cell epigenomics techniques have also been developed to investigate epigenetic features of the cellular components within heterogenous tissues, such as single-cell DNA methylation sequencing and single-cell chromatin mapping (Schwartzman and Tanay, 2015), providing an opportunity to identify epigenetic regulation patterns and characterize epigenetic heterogeneity. For example, single-cell DNA methylation sequencing revealed that tumor-derived clonal organoids from different colorectal cancer patients show different epigenetic states, and one tumor encompasses multiple epigenetic states (Roerink et al., 2018). Furthermore, combinational single-cell RNA, DNA and methylation sequencing has been used to study the heterogeneity of hepatocellular carcinoma (Hou et al., 2016). In addition, single-cell ChIP-seq was employed to investigate the heterogeneity of chromatin states in breast cancer, which revealed that drug-resistant tumors show more heterogeneity than sensitive tumors. Notably, a small population of tumor cells with resistance signatures could also be detected in the sensitive tumor, indicating the pre-existence of drug resistant subpopulations (Grosselin et al., 2019).

Recently, single-cell sequencing assay for transposase-accessible chromatin (scATAC-seq) has also been employed to dissect the tumor heterogeneity (Wang et al., 2019; LaFave et al., 2020). For example, scATAC-seq identified three cancer cell subpopulations in glioblastoma, including pro-neural, mesenchymal and intermediate cell states (Wang et al., 2019). Moreover, dynamic evolution of cancer cells in a mouse model of lung adenocarcinoma has been investigated by scATAC-seq, which revealed an epigenetic continuum of cancer progression, characterized by loss of cellular identify and progression to a metastatic state (LaFave et al., 2020). Together, these findings indicate that single-cell epigenomics also have advantages to exploring tumor heterogeneity and identifying mechanisms underlying cancer cells evolution.

## Dissecting Heterogeneity of Stromal Cells

Tumor microenvironment (TME) plays essential roles in cancer development and progression, which is composed of many types of cellular components and extracellular matrix (Hinshaw and Shevde, 2019). Importantly, single-cell sequencing has also been used to investigate the cellular heterogeneity of TME (Kieffer et al., 2020; Qian et al., 2020; Zhang et al., 2020). For example, a single-cell analysis of pan-cancer revealed a wide range of heterogeneity of stromal cells, including cancer-associated fibroblasts (CAFs), infiltrated immune cells, and endothelial cells (Qian et al., 2020). Moreover, a subset of FAP+ CAF could be further divided into eight subpopulations in Breast Cancers by scRNA-seq. Importantly, one CAF subpopulation characterized by high expression of genes encoding extracellular matrix proteins was revealed to drive immunotherapy resistance by increasing the protein levels of PD-L1 and CTLA4 in Treg cells through cell crosstalk (Kieffer et al., 2020). Similarly, scRNA-seq identified six CAF subpopulations in human intrahepatic cholangiocarcinoma, which could promote tumor progression by interacting with tumor cells (Zhang et al., 2020).

Furthermore, single-cell profiling of myeloid cells has been investigated in glioblastoma across species, which revealed that there are two distinct populations of tumor-associated macrophages, microglia- and monocyte-derived macrophages, which exist in the TME and compete for space (Pombo Antunes et al., 2021). Similarly, single-cell profiling of infiltrated T cells has been performed in multiple cancers (Zheng et al., 2017; Azizi et al., 2018; Li et al., 2019). For instance, scRNA-seq identified that a large number of CD8+ T cells exhibit continuous progression from an early effector state to dysfunctional T cell state in melanoma. Interestingly, this study also demonstrated that the dysfunctional CD8+ T cells are the major proliferating immune cells showing highly clonal and differentiating properties (Li et al., 2019). Moreover, scRNA-seq has been used to elucidate the heterogeneity of immune cells in treatment response to anti-PD1 in breast cancer, which revealed that PD1+ T cells undergo clonal expansion upon anti-PD1 treatment (Bassez et al., 2021). In addition, scRNA-seq has been employed to determine the heterogeneity of endothelial cells (ECs) in lung cancer, which identified 17 known and 16 unrecognized phenotypes of ECs (Goveia et al., 2020). Similarly, the subpopulations of ECs in tumors and their changes in gene expression following antiangiogenic treatment were analyzed by scRNA-seq (Zhao et al., 2018). Together, these studies demonstrated that single-cell technology greatly accelerates the understanding of stromal heterogeneity, providing new avenues to target these cellular components for precision cancer therapy.

## Tracing Cancer Cell Evolution by Single-Cell Sequencing

Cancer cell evolution is a fundamental process during tumor progression (Black and McGranahan, 2021). Single-cell technologies have been used to trace the dynamic evolution of cancer cells (Wang et al., 2014; Davis et al., 2020; Ireland et al., 2020; Schlesinger et al., 2020; Liu R. et al., 2021; Su et al., 2021). For example, it has been considered that acinar metaplasia is the first step during pancreatic ductal adenocarcinoma tumorigenesis. However, using scRNA-seq and trajectory analysis, Schlesinger et al. found that acinar cells and early metaplastic cells show a continuous change to one of two fates, tumorigenic or stomach metaplastic, suggesting that metaplastic cells may not be involved in the evolution process from acinar cells, early metaplastic cells to tumor cells (Schlesinger et al., 2020). Moreover, small cell lung cancer (SCLC) has been classified into four molecular subtypes, including ASCL1+, NEUROD1+, POU2F3+ and YAP1+ SCLC (Rudin et al., 2019). However, the cellular origins of these SCLC subtypes are still elusive. Through time-series scRNA-seq analysis, Trudy Oliver and colleagues demonstrated that MYC can drive the dynamic evolution of SCLC subtypes, promoting a temporal shift from ASCL1+ to NEUROD1+ and YAP1+ SCLC states (Ireland et al., 2020).

In order to trace the clonal evolution of cancer cells from primary tumor to metastatic tumor, Davis et al. examined the heterogeneity of primary tumors and early metastases of triple-negative breast cancer by the scRNA-seq. They found that the heterogeneity of metastatic tumors is consistent with that of primary tumors, but the proportion of a subpopulation obviously increases in metastatic tumors, indicating an enrichment of this subpopulation during the process of metastasis (Davis et al., 2020). Moreover, clonal evolution of breast cancer has been investigated through the scDNA-seq, which found that chromosome rearrangements occur in the early stage of tumor evolution whereas point mutations evolve gradually over the long-term, generating extensive clonal diversity (Wang et al., 2014). Furthermore, scDNA-seq has been used to study genomic heterogeneity and clonal evolution of gastroesophageal junction cancer, which found that the similarity between lymph node metastasis and primary tumor is greater than that between different lymph node metastases, indicating that different lymph node metastases can originate from the same primary tumor but evolve independently (Duan et al., 2021). Similarly, Su et al. investigated the clonal evolution of liver cancer by scRNA-seq and scDNA-seq (Su et al., 2021). In addition, through examining genomic alterations of primary colorectal cancer tumor cells and CTCs from the same patient, Gao et al. revealed convergent evolution of copy number alterations from primary to circulating tumor cells (Gao et al., 2017).

Drug treatments have been shown to drive cancer evolution and increase the intra-tumor heterogeneity (Eyler et al., 2020; Vander Velde et al., 2020; Cohen et al., 2021). For example, longitudinal scRNA-seq has revealed that there are three main trajectories of tumor clonal evolution in patients with multiple myeloma, indicating that nearly half of the patients show clonal dynamics and transcriptional changes. Notably, one patient showed the transition from clone 1 with high expression of CSAG1 and MS4A1 genes at baseline treatment to clone 2 with downregulated expression of CSAG1 and MS4A1 after 4 cycles of treatment (Cohen et al., 2021). Furthermore, scRNA-seq revealed dynamic phenotypic changes in the evolution of drug resistance in ALK positive NSCLC, and even short-term Alectinib exposure can significantly affect cell phenotypes, suggesting a drug-induced direct cellular adaptation (Vander Velde et al., 2020). Additionally, scRNA-seq has been used to trace the emergence of drug resistance in glioblastoma cells after treatment of RTK inhibitors, which revealed the critical roles of interplay between genetic and epigenetic mechanisms in drug resistance (Eyler et al., 2020).

## Hybrid Tumor Cell States Uncovered by Single-Cell Technologies

### Hybrid Epithelial/Mesenchymal Cells

By use of single-cell sequencing, multiple hybrid states of tumor cells have been identified in various cancers, such as hybrid epithelial/mesenchymal cells, hybrid tumor/immune cells and hybrid tumor/endothelial cells. These hybrid states could confer tumor cells with different potentials to adapt to the changing microenvironments. EMT has been recognized as an important cellular program not only in normal embryonic development but also in many diseases, especially cancer initiation and progression (Brabletz et al., 2018). Recently, cancer cell subpopulations with EMT feature have been identified in multiple cancers by single-cell sequencing. For example, glioblatoma cells have been classified into four subtypes by scRNA-seq, including neural proenitor-like (NPC-like), oligodendrocyte-progenitor-like (OPC-like), astrocyte-like (AC-like) and mesenchymal like (MES-like) cells. Importantly, a dynamic transition from OPC-like or NPC-like cells to MES-like cells was revealed, indicating a high plasticity of glioblastoma cells (Neftel et al., 2019). Moreover, using scRNA-seq and ST analysis, Ji et al. dissected the cellular composition and architecture of cutaneous squamous cell carcinoma. They found that a tumor-specific keratinocyte (TSK) subpopulation, expressing classic EMT markers, localizes to a fibrovascular niche and functions as a hub for intercellular communication (Ji et al., 2020).

In addition, Wouters et al. reported an intermediate state of melanocyte and mesenchymal cell, which was regulated by a set of transcription factors, including SOX6, NFATC2, EGR3, ELF1, and ETV4. They also demonstrated that knockdown of the SOX10 gene is sufficient to switch melanocytic and intermediate cell state to mesenchymal-like cell state (Wouters et al., 2020). Notably, the cell origins of CAFs are still elusive. One of the cell origins has been proposed is that CAFs can be derived from tumor cells undergone a EMT program, which can be distinguished by analyzing the genomic alterations (Sahai et al., 2020). In summary, by use of single-cell technologies, these studies indicated that a subset of tumor cells with EMT feature has been widely existed in heterogenous populations of multiple Cancers.

### Hybrid Tumor/Immune Cells

Immune Checkpoint Blockades (ICB) have been used in clinic to treat cancer patients. However, only a few patients respond to these ICBs. Unfortunately, the underlying mechanisms regarding immune evasion of tumor cells are largely unknown. Recently, a subpopulation of tumor cells expressing immune cell markers has been identified in several cancers by scRNA-seq. For example, Jin et al. identified a tumor cell population characterized by expression of epithelial-immune dual markers, such as classical epithelial marker EPCAM and immune markers, MHC-II and complement genes. The dual feature of tumor cells was observed to be positively correlated with the expression of co-inhibitory receptors on CD8^+^ T cells. Importantly, tumor cells with this dual feature exhibited a higher capacity for tumorigenesis and associated with poor prognosis of patients with nasopharyngeal carcinoma (Jin et al., 2020). Moreover, Miao et al. found that a subset of tumor-initiating stem cells in squamous cell carcinoma selectively express CD80, a previously identified immune cell surface ligand. They further demonstrated that CD80 is necessary for the tumor-initiating stem cells to endure immune attack and CD80 could dampen the activity of cytotoxic T cells through directly engaging with CTLA4 (Miao et al., 2019). Consistently, Wang et al. identified that cancer stem cells can upregulate another immune checkpoint molecule CD276 (B7–H3) in order to evade host immune attack. They found that CD274 is highly expressed by cancer stem cells of mouse and human head and neck squamous cell carcinoma, and anti-CD276 could eliminate these stem cells (Wang C. et al., 2021). Additionally, Chen et al. found that luminal prostate cancer cells express T-cell co-stimulatory genes, suggesting a potential role of tumor cells involved in antigen presentation (Chen et al., 2021). Taken together, these findings indicated that tumor cells expressing immune cell markers is one of the mechanisms by which tumor cells evade immunosurveillance, providing a new avenue for the development of immune checkpoint inhibitors and combined targeted therapy.

### Hybrid Tumor/Endothelial Cells

Angiogenesis is one of the cancer hallmarks. It has been reported that tumor cells could transdifferentiate into endothelial cells and form vascular mimicry in order to feed rapidly growing tumors (Maniotis et al., 1999; Kirschmann et al., 2012). Recently, single-cell sequencing has been used to understand the tumor angiogenesis. For example, Caroline Dive and colleagues found that a rare subpopulation of CTCs from SCLC patients co-expresses vascular endothelial-cadherin (VE-cadherin) and cytokeratin, which is consistent with the process of vasculogenic mimicry, a process during which tumor cells form endothelial-like vessels. They also found that knockdown of the VE-cadherin could increase sensitivity of SCLC cells to chemotherapy (Williamson et al., 2016). Consistently, a rare subpopulation of tumor-derived endothelial cells was observed to contribute to vessels within the tumor tissues in a mouse model of glioma (Carlson et al., 2021). In addition, Li et al. showed that disseminated melanoma cells could transdifferentiate into endothelial cells in intravascular niches of various metastatic organs (Li et al., 2020). Altogether, these findings indicated that tumor cells with endothelial cell features might play important roles in tumor growth, drug resistance and metastasis.

## Single-Cell Multi-Omics and Precision Cancer Therapy

Single-cell techniques have been applied in precision cancer therapy, such as tracing drug treatment responses and identifying novel therapeutic targets (Su et al., 2017; Yang et al., 2019; Jerby-Arnon et al., 2021). For example, melanoma cells with BRAF mutation often develope drug resistance after treatment with BRAF inhibitors. By use of single-cell functional proteomics, the activation of MEK/ERK and NFκB p65 pathways were revealed shortly after BRAF inhibition and before the emergence of drug resistance, suggesting that combining MEK and NFκB p65 inhibition with BRAF inhibitor could delay the adaptive cell state transition and development of resistant phenotypes (Su et al., 2017). Besides, HES6 was identified as a driver of metastasis in primary uveal melanoma by scRNA-seq, suggesting that HES6 may represent an actionable target of this tumor (Pandiani et al., 2021).

Moreover, single-cell techniques have been used to simultaneously determine the responses of heterogeneous tumors to multiple chemotherapeutic drugs, which could uncover the transcriptome networks underlying drug responses at single-cell resolution, and help to eliminate the effects of intra-tumoral heterogeneity on treatments (Roider et al., 2020; Srivatsan et al., 2020). Furthermore, single-cell techniques have advantages to identifying rare cancer cell subpopulations, which lead to the tumor progression and failure of cancer treatments (Kim et al., 2016; Miao et al., 2019; Prieto-Vila et al., 2019; Lee et al., 2020; Sehgal et al., 2021). For instance, scRNA-seq identified a subset of tumor-initiating stem cells in squamous cell carcinoma, which selectively express CD80 molecule and bind to the CTLA4 on cytotoxic T cells and thus damage the activity of T cells. Accordingly, blocking the binding of CTLA4 and CD80 could specifically eliminate these tumor-initiating stem cells and inhibit tumor relapse after immunotherapy (Miao et al., 2019). Besides, scRNA-seq revealed coexistence of multiple tumor cell subpopulations in metastatic renal cell carcinoma, whereas each tumor cell subpopulation showed distinct dysregulated signal pathways. This study further demonstrated that combinational inhibition of both EGFR and SRC signaling pathways could significantly enhance the therapeutic effect, indicating that single-cell sequencing can be used to optimize the strategy of targeted therapy (Kim et al., 2016). Similarly, scRNA-seq has been used to identify therapeutic targets for patients with refractory cancer (Lee et al., 2020).

In addition, single-cell techniques have been applied in identifying biomarkers to predict prognosis of cancer patients. For instance, by integrating large-scale bulk multi-omics and single-cell transcriptomic data of primary melanoma, a predictive model was constructed and 17 genes associated with the poor prognosis of patients were identified (Song et al., 2021). Finally, single-cell sequencing has been used to reveal the prognostic roles of stromal cell heterogeneity in multiple Cancers (Savas et al., 2018; Dominguez et al., 2020; Zhang Y. et al., 2021; Gong et al., 2021). For example, Gong et al. performed scRNA-seq of 66,627 cells from 14 nasopharyngeal carcinomas (NPCs), which revealed the stromal dynamics and NPC-specific characteristics in the TME of NPCs. Notably, they found that the dynamic immune signatures correlate with patient prognosis, such as increased infiltration of plasma B cells, dendritic cells and macrophages associated with a good prognosis (Gong et al., 2021). Moreover, scRNA-seq uncovered a subpopulation of CD8^+^ memory T cells in breast cancer, which showed high expression of immune checkpoint molecules and effector proteins. Importantly, this subset of T cells was observed to be significantly associated with an improved survival of patients with early-stage triple-negative breast cancer (Savas et al., 2018). Furthermore, Zhang et al. performed scRNA-seq analysis of renal cell carcinomas (RCC), which revealed that a higher fraction of endothelial cells associates with better overall survival of patients. Moreover, two macrophage subpopulations (macrophage-A and macrophage-B) were identified in the RCCs, high expression of the macrophage-A signature was observed to be associated with poor prognosis whereas high expression of the macrophage-B signature correlated with favorable prognosis (Zhang Y. et al., 2021). Besides, Dominguez et al. investigated the single-cell atlas of CAFs in pancreatic cancer by scRNA-seq, which revealed that a LRR15+ CAF subpopulation associates with a poor outcome of cancer immunotherapy (Dominguez et al., 2020). Taken together, single-cell technologies have shown great advantages in personalized therapy and prognosis prediction.

## Limitations of Single-Cell Technologies

Although single-cell technologies have greatly enhanced our understanding of the tumor heterogeneity, there are still multiple limitations of these techniques, such as limited sensitivity, scale and accuracy, which need to be addressed by technological improvements or combined with other technologies (Lei et al., 2021). Furthermore, most single-cell techniques performed analysis on dissociated cells, which could not interrogate spatial architecture of tumor tissues. With the advance of new techniques, such as the spatial transcriptomics (ST), this issue can be partly solved. However, the resolution of the current ST platform is still low, the capture spot usually contains a couple of cells (Wu et al., 2021). Besides, the transcriptome and proteome of cells could be disturbed during the preparation of single-cell suspension. Moreover, most scRNA-seq approaches only detected protein-coding genes by capturing polyA RNAs, which excluded all the non-coding genes. In addition, interpretation of data generated from single-cell omics techniques has been a challenge, which heavily depended on bioinformatics methods. However, each bioinformatic algorithm has its own advantages and limitations. For example, the number of cell types identified within a tumor could be affected by using different parameters. Finally, the cost of current single-cell omics techniques is extremely expensive compared with the bulk omics approaches.

## Conclusions

The dynamic evolution of tumor cells and their interactions with non-tumor cell components in the TME contributed to the tumor progression. Understating the heterogeneity of cellular compositions and their crosstalk in the TME will accelerate the development of personalized therapies. Fortunately, Single-cell multi-omics have shown great advantages in the dissecting the intra-tumoral heterogeneity. Importantly, the identification of cancer cell plasticity and their regulators have enabled us to understand the molecular underpinnings of cancer cell evolution during tumor progression.
